# Using a Quality Improvement Initiative to Decrease Oxygen Toxicity among Mechanically Ventilated Children

**DOI:** 10.1097/pq9.0000000000000849

**Published:** 2025-11-21

**Authors:** Salim Aljabari, Ethan Gillett, Zach Settle, Esma Birisci, Abdallah Dalabih

**Affiliations:** From the * Department of Pediatrics, University of Arkansas for Medical Sciences, Little Rock, Ark.; † Respiratory Care Department, Arkansas Children’s Hospital, Little Rock, Ark.; ‡ Department of Econometrics, Bursa Uludağ University, Bursa, Turkey; § Department of Pediatrics, Driscoll Children’s Health System, Corpus Christi, Tex.

## Abstract

**Introduction:**

Excessive oxygen supplementation in critically ill children can lead to hyperoxia, resulting in systemic toxicity and worse outcomes. Despite evidence linking hyperoxia to adverse outcomes, the overuse of oxygen therapy remains a widespread practice. This quality improvement initiative aimed to reduce hyperoxia exposure among mechanically ventilated children in the pediatric intensive care unit at Arkansas Children’s Hospital, aligning with the Second Pediatric Acute Lung Injury Consensus Conference guidelines.

**Methods:**

A multidisciplinary team implemented interventions in 2 Plan-Do-Study-Act cycles. The first cycle focused on staff education and standardizing oxygen saturation (SpO_2_) goals (90%–97%) in electronic health records. The second cycle introduced a best practice advisory to alert bedside staff when SpO_2_ exceeded 97% with the fraction of inspired oxygen (FiO_2_) greater than 0.21, prompting FiO_2_ weaning. Hyperoxia was defined as SpO_2_ 98%–100% with FiO_2_ greater than 0.21. We collected hourly SpO_2_–FiO_2_ data pairs from mechanically ventilated patients and calculated hyperoxia rates monthly.

**Results:**

Baseline data (January 2021 through June 2022) showed an average hyperoxia rate of 54.8%. Following the first Plan-Do-Study-Act cycle, the rate decreased to 41.0%, and after best practice advisory implementation, it further dropped to 28%, sustaining this reduction for more than 12 months. Mortality and mechanical ventilation duration did not change significantly (11.7%–9.4%, *P* = 0.12; and 8.16–4.8 d, *P* = 0.11, respectively).

**Conclusions:**

Using quality improvement methodology and electronic health record–based clinical decision support tools, we successfully reduced hyperoxia rates among mechanically ventilated children in the pediatric intensive care unit. This initiative highlights the importance of standardized oxygen management and real-time staff reminders in improving care practices.

## INTRODUCTION

Oxygen supplementation, a common intervention in the pediatric intensive care unit (PICU), can cause systemic toxicity and worsen the outcome if provided in excess.^1^ Several studies in recent years have demonstrated an association between hyperoxia [supraphysiologic partial pressure of arterial oxygen (PaO_2_) due to excessive oxygen supplementation] and mortality among critically ill children.^2–7^ Oxygen toxicity can cause damage by forming reactive oxygen species and inducing an inflammatory response, which leads to cell death/apoptosis. Cellular damage from oxygen toxicity has been reported in nearly every tissue type.^2,3^

Although the optimal oxygen parameters to avoid hypoxemia-related adverse effects among critically ill patients remain unknown, the second international guidelines by the Pediatric Acute Lung Injury Consensus Conference recommend maintaining oxygen saturation (SpO_2_) less than 98%.^8^ Additionally, the recently published OxyPICU randomized controlled trial on liberal (>94%) versus conservative (88%–92%) SpO_2_ goals in critically ill and mechanically ventilated children supported the conservative SpO_2_ goals.^9^ The aim of this quality improvement (QI) initiative was to reduce hyperoxia exposure among critically ill and mechanically ventilated children at the Arkansas Children’s Hospital PICU.

## METHODS

### Context

The PICU at Arkansas Children’s Hospital is an academic 26-bed unit, caring for both medical and surgical patients, except those with critical cardiac conditions. It is the only PICU in the state and has an average of more than 1,700 admissions annually. On average, one-third of the PICU admissions receive invasive mechanical ventilation and oxygen supplementation. The PICU is staffed with faculty intensivists, pediatric critical care medicine fellows, and pediatric residents. Critical care nurses (registered nurses) and PICU core respiratory therapists (RTs) provide most of the care, though occasionally assisted by noncore RTs.

Historically, oxygen therapy and SpO_2_ goals were not standardized and have been provider-dependent. The PICU admission order set contained a modifiable, preselected SpO_2_ goal of 92%–100%. The fraction of inspired oxygen (FiO_2_) order on the mechanical ventilator order set includes a section addressing whether the bedside nurse can or cannot wean FiO_2_.

### Interventions

**Fig. 1.**
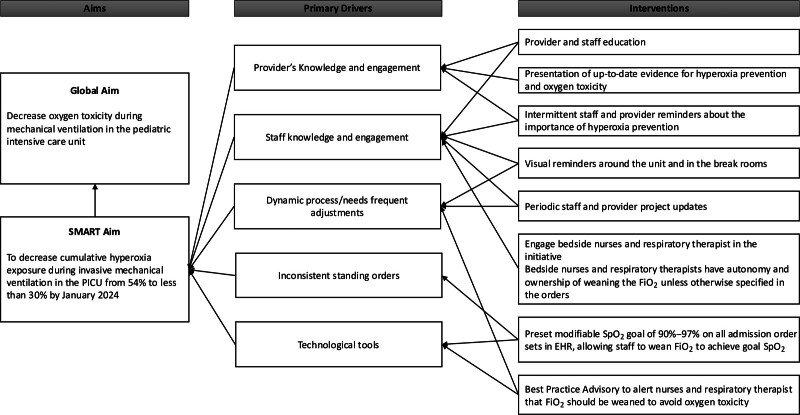
Key driver diagram.

To inform improvement interventions and key drivers, we identified a multidisciplinary team of PICU physicians, nurses, and RTs who championed this initiative. A summary of the key drivers and correlating interventions is presented in Figure 1.

We implemented several interventions to achieve the project goal. We grouped those interventions into 3 main categories outlined here. The first 2 represented the first Plan-Do-Study-Act (PDSA) cycle, and the third described the second PDSA cycle.

#### Intervention 1

The first intervention focused on faculty and staff knowledge and engagement. We delivered targeted education to physicians, advanced practice providers, bedside nurses, and RTs on the potential harms of hyperoxia and the significance of this QI initiative. We presented evidence for oxygen toxicity and for the association between hyperoxia and mortality. An educational module about oxygen toxicity and the importance of hyperoxia prevention was added to the mandatory annual online nursing curriculum. Additionally, visual reminders were presented in the unit’s common areas.

To foster provider and staff engagement, we organized a series of reminders to the team about the importance of hyperoxia prevention. Those reminders were distributed periodically via email lists, unit private social media groups, and the announcement monitors in the PICU common areas. We additionally provided bimonthly updates on progress during our PICU QI meetings, attended by most of the providers with representation from the nursing and respiratory therapy staff.

#### Intervention 2

Before the start of this project, the ventilator order in our electronic health records (EHRs) had 2 sections related to oxygen therapy. One section focused on SpO_2_ goals, and the other section focused on any limitations to FiO_2_ weaning. No default option was preselected in either section. The ordering provider had to choose 1 option or free-type as an alternative. To avoid missed opportunities, the ventilator order was changed to have the SpO_2_ goal preselected as 90%–97%, and the limitation section preselected to allow staff to wean the FiO_2_ toward 0.21 if the SpO_2_ was within range.

#### Intervention 3

The third intervention, which constituted the second PDSA cycle, used the EHR to remind the bedside staff to wean FiO_2_ when appropriate. We designed a best practice advisory (BPA) in the EHR (Epic Systems Corporation, Verona, WI) to alert the bedside nurses and RTs about the risk of hyperoxia and encourage them to wean FiO_2_ if appropriate. The BPA was designed to alert if the SpO_2_ was recorded as 98%–100% and the FiO_2_ recorded as greater than 0.21 at the same time. To prevent alarm fatigue, we limited the BPA alert to bedside nurses and RTs, restricting it to no more than once every 4 hours. Additionally, if the patient had pulmonary hypertension in the diagnosis or problem lists, this BPA would not trigger an alert.

### Measures and Data Collection

As most of the patients do not undergo frequent PaO_2_ evaluations, we used SpO_2_ values to define hyperoxia. As recommended by the Second Pediatric Acute Lung Injury Consensus Conference, SpO_2_ values greater than 97% can be associated with supraphysiologic PaO_2_ values and should be avoided.^8^ We defined hyperoxia as SpO_2_ of 98%–100% when FiO_2_ is greater than 0.21. We defined our primary outcome measure as the percentage of SpO_2_–FiO_2_ data pairs within the hyperoxia definition.

Hourly SpO_2_–FiO_2_ data pairs were collected on all patients admitted to the PICU who required mechanical ventilation. The rate of hyperoxia was calculated as the number of SpO_2_–FiO_2_ data pairs that met the hyperoxia definition divided by the total number of data pairs. Data were presented as a unit aggregate monthly.

A report of the monthly hyperoxia rate among mechanically ventilated patients in the PICU was added to the EHR PICU QI dashboard. Data from the 12 months before the project initiation were collected as a baseline. Patients receiving mechanical ventilation via tracheostomy tube were excluded from the data because a significant proportion of them were managed on home ventilators, with oxygen supplementation measured in liters per minute rather than FiO_2_. As balancing measures, we compared mortality, length of intensive care unit stay, and duration of mechanical ventilation pre- and postintervention.

Data to calculate the hyperoxia rate were extracted directly from the EHR. Data for mortality, length of stay, and duration of mechanical ventilation were obtained from the Virtual PICU System data registry.

#### Ethical Consideration

The University of Arkansas for Medical Sciences/Arkansas Children’s Hospital institutional review board evaluated the project and deemed it not human research.

### Analysis

We created statistical process control charts, specifically P-charts, for our outcome measure. We used standard Shewhart rules to shift the mean and control limits when a special cause variation was noted. A shift was noted when 8 or more points in a row were above or below the center line, and a trend was noted when 6 consecutive points were increasing or decreasing.^10^ We used QI Macros software 2024 (KnowWare International, Inc., Denver, CO) to create the control chart.

Pre- and postintervention data were compared using standard statistical analysis with paired *t* tests. We used the SPSS software to conduct the analysis. A *P* value of less than 0.05 was considered statistically significant.

## RESULTS

**Fig. 2.**
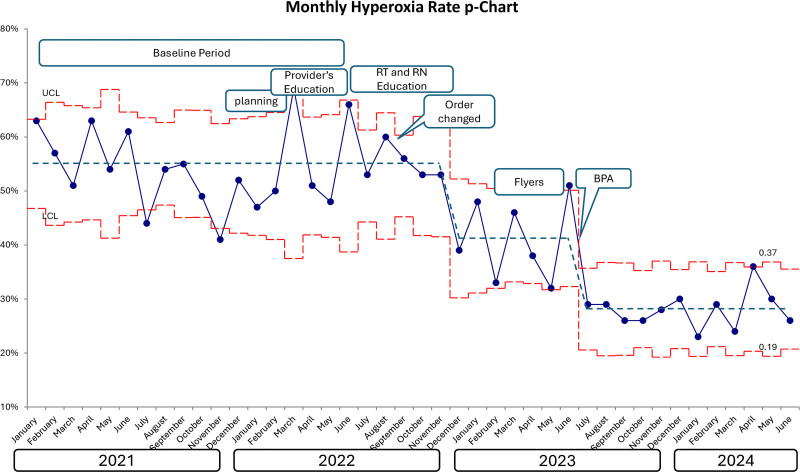
Statistical process control: P-chart. RN, registered nurse; LCL, lower control limit; UCL, upper control limit.

An annotated statistical process control chart of our primary outcome is shown in Figure 2. Both intervention 1 (awareness and engagement) and intervention 2 (default EHR orders) were part of PDSA cycle 1 and occurred over a few months.

Baseline data were from January 2021 to June 2022. During this period, 712 patients were admitted to the PICU and required mechanical ventilation. The average monthly hyperoxia rate was 54.8% throughout the baseline period. The average length of mechanical ventilation during the baseline period was 8.16 days (SD 18.8 d), and mortality was 11.7%. After implementing the first PDSA cycle, the average monthly hyperoxia rate dropped to 41.0%.

With the launch of the BPA, the average monthly hyperoxia rate dropped to 28% and was sustained for more than a year. During the 12 months after launching the BPA, there were 513 admissions that required mechanical ventilation. The average length of mechanical ventilation was 4.8 days (SD 6.1 d), and mortality was 9.4%.

Table 1 compares key clinical and demographic variables between subjects in the preintervention and postintervention periods. There were no significant differences in pediatric index of mortality III risk of mortality (6.7% versus 6.0%, *P* = 0.22) or mortality rates (11.7% versus 9.4%, *P* = 0.2). Median PICU length of stay and duration of mechanical ventilation remained stable across both periods, with no statistically significant differences.

**Table 1.** Comparison of Cohorts Pre- and Postintervention

Variable	Preintervention, January 1, 2021–June 30, 2022, N = 712	Postintervention, July 1, 2023–June 30, 2024, N = 513	*P*
Age, y, Avg (SD)	8.0 (7.9)	6.6 (6.7)	0.002
Female, n (%)	211 (41)	231 (45)	0.12
PIM III ROM	6.7 (14.9)	6.0 (14.0)	0.22
Mortality, n (%)	83 (11.7)	48 (9.4)	0.2
PICU LOS, Avg. (SD)	10.8 (17.9)	10.1 (14.2)	0.5
Duration of MV, Avg. (SD)	8.16 (18.8)	4.8 (6.1)	0.11

Avg., average; LOS, length of stay; MV, mechanical ventilation; PIM, pediatric index of mortality; ROM, risk of mortality.

## DISCUSSION

Using QI methodology, we decreased hyperoxia exposure among critically ill and mechanically ventilated children in our PICU. We decreased the average hyperoxia exposure from 54.8% to 28% and sustained this change for more than 12 months. Mortality and length of mechanical ventilation were not significantly different in the postintervention phase compared with the preintervention period.

The initial barrier we faced was a change in culture and practice. Although oxygen toxicity is not a new concept and has long been a concern among PICU practitioners,^11,12^ efforts have traditionally focused on avoiding FiO_2_ levels greater than 60% rather than addressing supraphysiologic PaO_2_ or SpO_2_ levels. The concept of systemic oxygen toxicity with supraphysiologic PaO_2_ due to oxygen supplementation less than 60% is a recent development.^13,14^ Our first intervention aimed to address this barrier, using a series of focused educational activities and presentations of evidence for the adverse effect of hyperoxia in the PICU. Providers direct oxygen therapy in the PICU, but for the most part, bedside nurses and RTs deliver it. Hence, it was essential to reach every team member, which posed several challenges.

From the provider’s perspective, in addition to the basic education, it was important to provide clear evidence of oxygen toxicity even with FiO_2_ levels lower than 60% and the benefit of preventing hyperoxia. At the same time, it was essential to address any potential balancing measures and exceptional patient conditions such as pulmonary hypertension. Given their large numbers and varied schedules for the bedside nursing staff, incorporating the education into their mandatory annual training module, along with using additional educational strategies and reminders, was essential to promote widespread understanding and adoption of the practice.

As we achieved better provider and staff knowledge, acceptance, and engagement, the hyperoxia rate improved from the baseline. However, we did not reach the goals. The bedside nurse and RT of a critically ill patient have numerous scheduled and rising tasks to accomplish. Oxygen weaning, though important, becomes a relatively small task among many competing priorities. Additionally, most patients with acute respiratory failure experience fluctuating respiratory dynamics, which leads to varying oxygenation parameters and FiO₂ requirements. Therefore, to avoid hyperoxia, bedside staff may need to frequently adjust FiO₂ to maintain appropriate SpO₂ levels while preventing both hypoxemia and hyperoxia.

To overcome some of those barriers, we implemented the clinical decision support tool.^15^ The BPA served as a reminder to bedside staff when SpO_2_–FiO_2_ values fell within the hyperoxia range, encouraging them to wean the FiO_2_. This intervention served 2 purposes. It reminded the busy bedside staff about this important task when missed, and it served as a constant reminder to the team about the importance of avoiding hyperoxia.

The BPA functioned as an effective reminder for staff, though it did not include a hard stop. Clinical decision support tools such as BPAs can be valuable in guiding practice, but they must be used thoughtfully to avoid contributing to alarm fatigue.^16^ In our experience, the BPA has also played a key role in raising awareness and supporting our efforts to reduce hyperoxia. We hope that, over time, hyperoxia prevention will become embedded in our clinical culture.

Reliability science offers a structured framework for understanding and improving the consistency of healthcare processes by categorizing interventions into levels of reliability—from level 1 (basic standardization and individual vigilance) to level 3 (system-level design that prevents errors altogether).^17^ Moving from lower to higher levels of reliability is essential for sustaining improvement and reducing dependence on individual performance.^17^ In our project, we initially used level 1 strategies such as staff education and protocol communication. However, the implementation of the BPA alert represented a critical shift toward level 2 reliability by embedding the desired action into the workflow through a system-level intervention. This design not only increased adherence but also made it easier to do the right thing and harder to omit critical steps, enhancing the long-term sustainability of our process improvements.

There are no specific recommendations on hyperoxia prevention; hence, it is unclear how low we should target our outcome measure.^18^ Additionally, there is a balance between hypoxia prevention and hyperoxia prevention. As patients with acute lung injury may experience dynamic and ever-changing oxygenation parameters, absolute avoidance of hyperoxia could be impossible.

Balancing metrics, namely the mortality and duration of mechanical ventilation, did not change significantly, highlighting the safety of hyperoxia prevention. As we continue to maintain low hyperoxia rates in our unit and gather a larger sample size, we hope to demonstrate improvements in mortality outcomes.

Our project and data have several limitations, some of which are inherent to the nature of data recording in the EHR. The current norm is to record vital signs and other important values at hourly rates, although some of those values, such as FiO_2_ and SpO_2_, change at much higher frequencies. One can argue that the hourly FiO_2_–SpO_2_ pair is a brief depiction of the oxygenation status and not a complete representation of it. However, the implemented interventions do not target those values but rather target the culture and practice of bedside staff and ordering providers. The hyperoxia rate we used was merely a marker of the practice. The significant improvement we present reflects the change in the practice and engagement of providers and staff in preventing hyperoxia.

Additionally, an SpO_2_ of 98%–100% is a substitute marker for hyperoxia. Most of the patients did not undergo serial PaO_2_ evaluation.^19,20^ Therefore, to achieve an impactful change, a noninvasive alternative is needed. Although some patients with an SpO_2_ between 98% and 100% might not be experiencing hyperoxia, many would be. This circumstance is reflected in the recently published pediatric randomized controlled trial of conservative versus liberal SpO_2_ goals in mechanically ventilated patients. Another limitation is related to the variation in pulse oximetry accuracy across different skin pigmentation tones.^21^ Finally, we were unable to capture data on individual episodes of hypoxemia. We therefore could not determine whether the frequency of such episodes was impacted by this QI initiative, due to technical challenges in tracking them. Future iterations of this initiative could focus on addressing this limitation.

This effort was multidisciplinary and required a cultural shift. The automated report was a cornerstone in the success of the project. It allowed real-time data monitoring with no manual effort. Our next steps will include refining the BPA to prevent it from triggering if the FiO_2_ has already been weaned since the last recorded. We also intend to expand the hyperoxia prevention initiative to include all patients, regardless of the type of respiratory support they are receiving. Additionally, we aim to further investigate how the hyperoxia prevention improved some of the patient outcome measures.

## CONCLUSIONS

Using standard QI methodology, we successfully achieved sustained lower hyperoxia rates among critically ill and mechanically ventilated children. Using EHR clinical decision support tools, we achieved a more than 30% decrease in hyperoxia rate, with significantly less variation than baseline.

## ACKNOWLEDGMENTS

Preliminary data from this study were accepted for presentation at the SCCM Congress 2025, Orlando, Florida, but were not presented.
